# Explicit and implicit effects of gaming content on social media on the behavior of young adults

**DOI:** 10.3389/fpsyg.2024.1332462

**Published:** 2024-01-24

**Authors:** Daisuke Jitoku, Nanase Kobayashi, Yuka Fujimoto, Chenyu Qian, Shoko Okuzumi, Shisei Tei, Daisuke Matsuyoshi, Takehiro Tamura, Hidehiko Takahashi, Takefumi Ueno, Makiko Yamada, Junya Fujino

**Affiliations:** ^1^Department of Psychiatry and Behavioral Sciences, Graduate School of Medical and Dental Sciences, Tokyo Medical and Dental University, Tokyo, Japan; ^2^Institute for Quantum Life Science, National Institutes for Quantum Science and Technology, Chiba, Japan; ^3^Department of Psychiatry, Nara Medical University, Nara, Japan; ^4^Department of Psychiatry, Graduate School of Medicine, Kyoto University, Kyoto, Japan; ^5^Medical Institute of Developmental Disabilities Research, Showa University, Tokyo, Japan; ^6^Institute of Applied Brain Sciences, Waseda University, Saitama, Japan; ^7^School of Human and Social Sciences, Tokyo International University, Saitama, Japan; ^8^Center for Brain Integration Research, Tokyo Medical and Dental University, Tokyo, Japan; ^9^Division of Clinical Research, National Hospital Organization, Hizen Psychiatric Medical Center, Saga, Japan; ^10^Department of Functional Brain Imaging, Institute for Quantum Medical Science, National Institutes for Quantum Science and Technology, Chiba, Japan; ^11^Division of the Humanities and Social Sciences, California Institute of Technology, Pasadena, CA, United States

**Keywords:** cognitive flexibility, cue reactivity, gaming addiction, implicit association test, self-efficacy

## Abstract

Excessive gameplay can have negative effects on both mental and physical health, especially among young people. Nowadays, social media platforms are bombarding users with gaming-related content daily. Understanding the effect of this content on people’s behavior is essential to gain insight into problematic gaming habits. However, this issue is yet to be studied extensively. In this study, we examined how gaming-related content on social media affects young adults explicitly and implicitly. We studied 25 healthy young adults (average age 21.5 ± 2.2) who played online games casually and asked them to report their gaming desire. We also conducted an implicit association test (IAT) to measure their implicit attitudes toward gaming-related content. We also investigated the relationship between these measures and various psychological factors, such as personality traits, self-efficacy, impulsiveness, and cognitive flexibility. The results revealed that participants had a higher explicit gaming desire when exposed to gaming-related cues on social media than neutral cues. They also had a robust positive implicit attitude toward gaming-related content on social media. Explicit gaming desire was positively correlated with neuroticism levels. Furthermore, the IAT effect was negatively correlated with self-efficacy and cognitive flexibility levels. However, there were no significant correlations between explicit gaming desire/IAT effect and impulsiveness levels. These findings suggest that gaming-related content on social media can affect young adults’ behavior both explicitly and implicitly, highlighting the need for further research to prevent gaming addiction in vulnerable individuals.

## 1 Introduction

Online games have grown immensely popular over the past few decades, both as recreation and for specific applications such as mental, physical, and cognitive training (Sailer and Homner, 2020; Dever et al., 2022; Han et al., 2022; Aydin and Kuş, 2023). These games can promote relaxation by allowing participants to leave their regular duties, and experience fantastical environments (Sailer and Homner, 2020; Maldonado-Murciano et al., 2022). Despite these benefits, online gameplay can become an obsession that replaces human interactions, and it may even impair mental and physical health, which has attracted widespread public concern and clinical attention, particularly among young generations (King et al., 2020; Zhang et al., 2020; Gülü et al., 2023).

Recently, social media, such as YouTube, Twitter, and Instagram, are increasingly used for communication, learning, and collaboration (Carr and Hayes, 2015; Kuss and Griffiths, 2017). In line with this trend, people are exposed to excessive gaming-related content on social media in daily life (Balakrishnan and Griffiths, 2017). They connect with their gaming community through social media platforms, enjoy watching others play games, and learn how to become better at games. A better understanding of the effects of gaming-related content on social media can offer additional insights into the psychological mechanisms of problematic game use and practical intervention strategies.

The psychocognitive model suggests that the addiction-related cues would trigger positive expectations, which drive the addictive behavior (Hyman et al., 2006; Starcke et al., 2018). Accordingly, a number of experimental studies have investigated reactions to gaming cues, which provide useful information for the pathogenesis of excessive gameplay (Liu et al., 2017; Kim et al., 2018; Ma et al., 2019). However, most previous studies used pictures or videos of familiar games as stimuli, without contextual information; thus, reaction to context-relevant gaming content on social media remains largely unknown. A behavioral study using real-world gaming-related cues on social media should improve ecological validity and expand the findings of previous studies of gaming cue reactivity.

Previous studies have used explicit self-report to evaluate the effects of gaming cues. However, self-reported responses to addictive content predict only some variabilities in future behavior (Webb and Sheeran, 2006; Garrison et al., 2018). Additional variability in behavior might be explained by implicit processes inaccessible to conscious awareness but not captured by self-report (Falk et al., 2010; Garrison et al., 2018). Gaming-related content on social media are pervasive environmental cues that people attend to both consciously and unconsciously (Balakrishnan and Griffiths, 2017). Thus, the effects of gaming-related cues on social media should be assessed explicitly and implicitly to get a broader picture of them.

To date, several factors are known to influence gaming addiction. In particular, personality traits, self-efficacy, impulsivity, and cognitive flexibility (CF) are repeatedly associated with individual differences in vulnerability to problematic game use. Neuroticism was identified as an essential factor influencing problematic online gaming (Chu et al., 2022). Chung et al. (2020) showed significant differences in self-efficacy according to the extent of gaming. Individuals that are more impulsive spend more time playing video games (Gentile et al., 2012). Regarding CF, recent studies have found an association between reduced CF and the severity of gaming addiction in individuals with internet gaming disorder (IGD) (Weinstein et al., 2017; Yang X. et al., 2023). Furthermore, these factors have been reported to play important roles in cue reactivity. For example, Glautier et al. (2000) showed that the levels of neuroticism and extraversion were associated with alcohol cue reactivity. A strategy focused on self-efficacy reportedly reduces cue-induced craving for addictive behaviors such as smoking (Loeber et al., 2006; Ono et al., 2018). Previous studies found that impulsivity moderated the relationship between food cue reactivity and food intake (van den Akker et al., 2014). Cocaine-dependent individuals are reportedly vulnerable to impairments in CF and inhibition in the face of stress and cue reactivity (Milivojevic et al., 2017). Thus, investigating the relationship between cue reactivity and these factors is vital to detect individuals susceptible to gaming-related content on social media.

**Figure 1:**
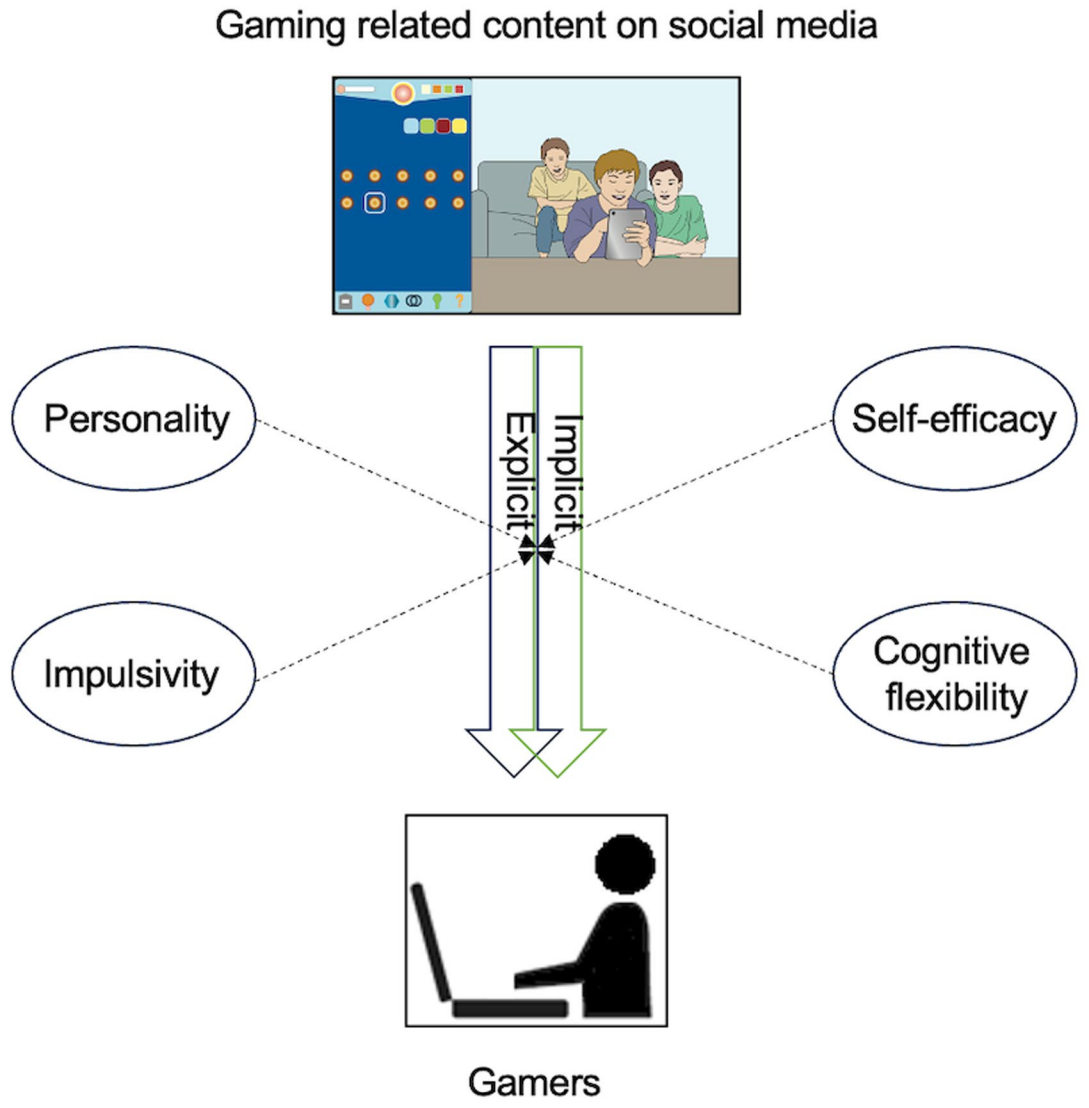
Study’s hypothetical framework.

This study investigated the explicit and implicit effects of gaming-related content on social media among young adults. For explicit measures, self-reported cue-induced desire for gaming was estimated. Regarding implicit measures, the implicit association test (IAT) was employed based on previous studies (Ames et al., 2014; Snagowski et al., 2015; Roh et al., 2018). We hypothesized that gaming-related content on social media significantly influenced participants’ behavior both explicitly and implicitly and that levels of cue reactivity were significantly correlated with the abovementioned factors influencing gaming addiction and cue reactivity. The hypothetical framework diagram is depicted Figure 1.

## 2 Methods

### 2.1 Participants

The study enrolled 26 healthy volunteers aged between 19 and 25 who played online games casually. Only male participants were selected because of a sex difference in the mechanism for gaming addiction, and men have higher gaming addiction potential than women (Dong et al., 2018, 2019; Wang et al., 2022). One participant faced a technical error during data collection, so data obtained from 25 participants were analyzed. All participants played online games regularly for at least an hour weekly and did not meet the IGD criteria proposed by the Diagnostic and Statistical Manual of Mental Disorders 5th Edition (DSM-5). They also did not have any psychiatric disorders, history of head trauma, serious medical or surgical illness, or substance abuse. Additionally, none of the participants were current smokers. To estimate the participants’ levels of gameplay, weekly gaming time and gaming history were assessed based on previous studies (Dong et al., 2018, 2019). In addition, based on previous studies (Dong et al., 2018, 2019), all participants completed the internet addiction test (Young, 1998; Ishii et al., 2023) to measure the degree of dependence on the internet. Predicted IQ was estimated using the Japanese version of the National Adult Reading Test short form (Matsuoka et al., 2006). Details are described in the Supplementary methods.

This study was approved by the institutional review board of Tokyo Medical and Dental University Hospital and was conducted in accordance with the Code of Ethics of the World Medical Association. After explaining the entire study, written informed consent was obtained from all participants.

### 2.2 Behavioral tasks

#### 2.2.1 Explicit desire task

To estimate the participants’ explicit gaming desire, we designed the EDT based on the methodologies in the previous studies of substance and behavioral addiction (Vollstädt-Klein et al., 2011; Courtney et al., 2014; Liu et al., 2017; Dong et al., 2019). Sixteen gaming-related videos such as persons enjoying a game, introducing a game, and teaching how to capture a game from social media platforms were selected. A game in each video was different from each other. The 16 games featured in the videos were all popular online games (shooting games, role-playing games, puzzle games, or sports games) in Japan. Sixteen neutral videos (nongaming videos, such as furniture-, hygiene-, travel-, and work-related videos) were also selected from social media platforms. Based on the previous studies (Tapert et al., 2003; Vollstädt-Klein et al., 2011; Chen Y. et al., 2018), they were chosen to match as much as possible each gaming-related video on complexity, content, design, luminance, color, action, and presence of faces. Details are described in Supplementary methods.

**Figure 2:**
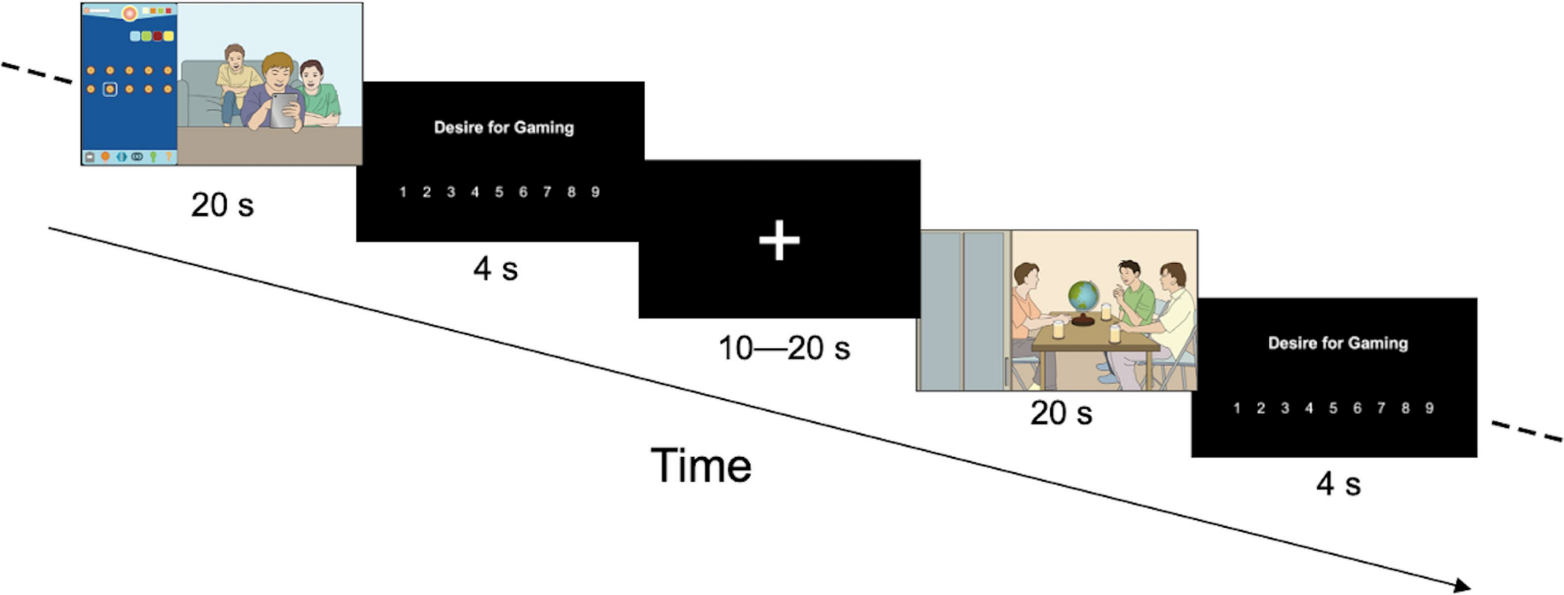
Explicit desire task (EDT). After each video was presented, participants were asked to rate the gaming desire from 1 (no desire) to 9 (extreme desire).

The videos were displayed pseudo-randomly, and after each video was presented, the participants were asked to rate the gaming desire from 1 (no desire) to 9 (extreme desire) (Figure 2). The experiment was conducted using E-Prime (Psychology Software Tools, Inc., Pittsburgh, PA, USA). The levels of participants’ explicit gaming desire were computed as follows: mean scores of the gaming desire in gaming-related videos–those in neutral ones. Higher scores denote higher levels of explicit gaming desire.

#### 2.2.2 IAT

In this study, the IAT (Greenwald et al., 1998, 2003) was modified with gaming-related pictures on social media. Previous studies have revealed that the IAT reliably predicts addictive behaviors (Ames et al., 2014; Snagowski et al., 2015; Roh et al., 2018).

**Figure 3:**
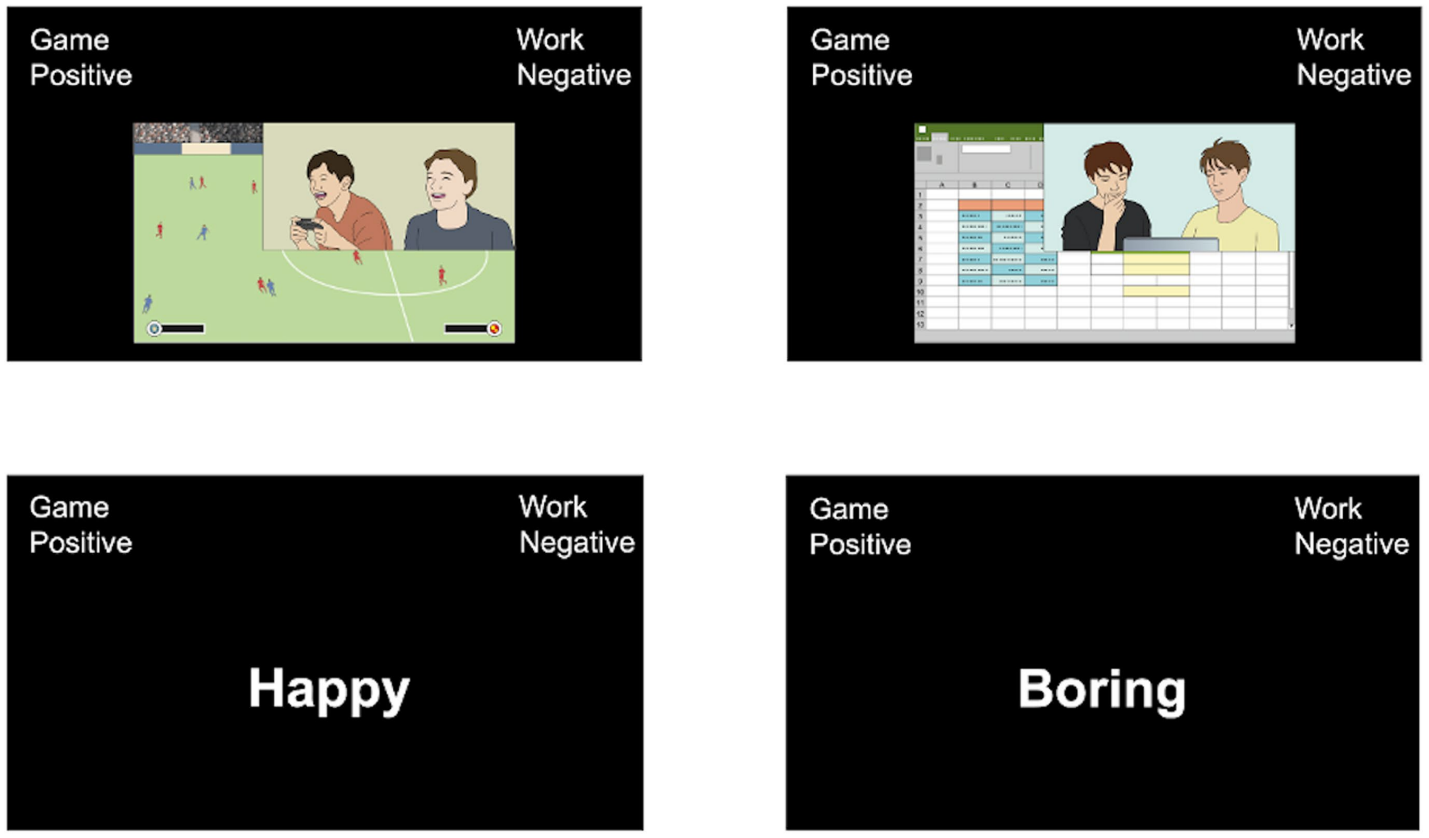
Implicit association test (IAT). Here, participants are asked to classify stimuli that appear at the lower portion of a computer screen into corresponding categories and pair attributes appearing in the screen’s upper left or right corners.

To assess individual differences in the levels of implicit positive attitudes toward gaming-related content on social media, the IAT evaluated the extent to which participants associated game vs. work (target categories) with positive vs. negative (attribute categories) (Figure 3). Four items were selected as the to-be-sorted stimuli for each concept and the present IAT comprised seven blocks according to the standard procedures (Greenwald et al., 1998, 2003; Fujino et al., 2017; Tei et al., 2018). Similar to the EDT, four gaming-related pictures were captured from the social media platforms for the target concept of game. The 4 games featured in the pictures were all popular online games (shooting games, role-playing games, puzzle games, or sports games) in Japan. For the target concept of work, four work-related pictures were also selected from social media platforms. They were selected to match each gaming-related picture on complexity, content, design, luminance, color, action, and presence of faces as much as possible. Stimuli from the categories positive/negative were happy, fun, attractive, and excited, and painful, sad, difficult, and boring, respectively. Participants were asked to classify stimuli that appeared at the lower portion of a computer screen into corresponding categories and paired attributes appearing in the upper left or right of the screen. They were told to be as accurate and as quickly as possible. The IAT was conducted using the E-Prime software (Psychology Software Tools, Inc.).

The IAT effect scores were computed based on the improved scoring algorithm (Greenwald et al., 2003). High scores represented quicker associations of game–positive and work–negative (congruent condition) relative to game–negative and work–positive (incongruent condition); thus, higher scores indicate higher levels of implicit positive attitudes toward gaming-related content on social media. Details are described in Supplementary methods.

### 2.3 Familiarity ratings

Following the tasks, the participants were asked to rate the familiarity of each game presented in the EDT and IAT from 1 (very unfamiliar) to 9 (very familiar).

### 2.4 Psychological measures

To estimate personality traits, self-efficacy, impulsivity, and CF, we administered the NEO-Five Factor Inventory (NEO-FFI) (Costa and McCrae, 1992; Shimonaka et al., 1998), self-efficacy scale (Bandura, 2006; Ono et al., 2018), Barratt Impulsiveness Scale, 11th version (BIS-11) (Patton et al., 1995; Someya et al., 2001), and CF scale (Martin and Rubin, 1995; Oshiro et al., 2016; Fujino et al., 2019; Sami et al., 2023), respectively. Higher scores indicate higher levels of each factor. Details are described in Supplementary methods.

### 2.5 Statistical analyses

Statistical analyses were performed using IBM SPSS Statistics version 24 (IBM Corp., Armonk, NY, USA). As mentioned above, explicit gaming desire and IAT effect were estimated. Correlational analyses between these measures and participants’ characteristics/psychological measures [age, IQ, internet addiction test, weekly gaming time, gaming history, NEO-FFI (neuroticism, extraversion, openness, agreeableness, and conscientiousness), self-efficacy, BIS-11, and CF scale] were performed. First, we conducted Shapiro–Wilk tests to estimate the normality of measures (non-normality determined at *p* < 0.05). Following the previous studies (Solas et al., 2013; Ferreira et al., 2021), correlation analysis was performed using Pearson or Spearman based on the normality of variables. Results were considered statistically significant at *p* < 0.05 (two-tailed).

## 3 Results

The characteristics of the participants are presented in Table 1. According to the internet addiction test, no participants showed severe symptoms.

**Table 1:** Participants’ demographics.

	Total (*n* = 25)
Age (years, Mean ± SD)	21.5 ± 2.2
Predicted full-scale IQ (Mean ± SD)	108.5 ± 7.4
Internet addiction test (Mean ± SD)	33.5 ± 8.3
Weekly gaming time (hours, Mean ± SD)	5.7 ± 5.4
Gaming history (years, Mean ± SD)	10.1 ± 3.3
NEO-FFI	
Neuroticism (Mean ± SD)	20.4 ± 9.5
Extraversion (Mean ± SD)	27.8 ± 7.0
Openness (Mean ± SD)	31.2 ± 7.2
Agreeableness (Mean ± SD)	31.1 ± 5.2
Conscientiousness (Mean ± SD)	29.6 ± 7.2
Self-efficacy (Mean ± SD)	78.4 ± 15.7
BIS-11 (Mean ± SD)	64.2 ± 10.2
CF scale (Mean ± SD)	51.4 ± 7.0

BIS-11, Barratt Impulsiveness Scale, 11th version; CF, cognitive flexibility; NEO-FFI, NEO-Five Factor Inventory.

The participants exhibited higher explicit gaming desire by the gaming-related cues on social media than the neutral ones (game 4.09 ± 1.52, neutral 1.63 ± 0.87, *p* < 0.01). Regarding the IAT, the mean response latency for incongruent condition (game–negative and work–positive) was significantly longer than that for congruent condition (game–positive and work–negative) (congruent 610.2 ± 88.1 ms, incongruent 1090.4 ± 156.8 ms, *p* < 0.01), which indicated that the participants showed robust and positive implicit attitudes toward gaming-related cues on social media. Familiarity rating scores were not significantly correlated with an explicit gaming desire (Pearson’s *r* = 0.24, *p* = 0.24) or the IAT effect (Pearson’s *r* = 0.26, *p* = 0.21), respectively.

**Figure 4:**
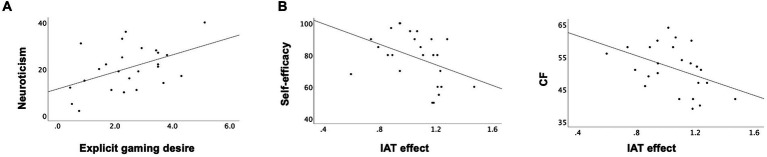
Correlations of explicit gaming desire and IAT effect with psychological measures. **(A)** Correlations between explicit gaming desire and neuroticism (*r* = 0.48, *p* = 0.02). **(B)** Correlations between IAT effect and self-efficacy (*r* = −0.41, *p* = 0.04) and CF (*r* = −0.43, *p* = 0.03). CF, cognitive flexibility; IAT, implicit association test.

We did not find any significant correlations between explicit gaming desire/IAT effect and internet addiction tests or weekly gaming hours (Table 2). However, the explicit gaming desire positively correlated with the levels of neuroticism (Pearson’s *r* = 0.48, *p* = 0.02) (Figure 4A). In addition, the IAT effect negatively correlated with the self-efficacy (Pearson’s *r* = −0.41, *p* = 0.04) and the CF (Pearson’s *r* = −0.43, *p* = 0.03) scales, respectively (Figure 4B). That is, the participants with lower levels of self-efficacy or CF abilities showed higher IAT effect. There were no significant correlations between the explicit gaming desire and impulsivity levels (Pearson’s *r* = 0.36, *p* = 0.08) or the IAT effect and impulsivity levels (Pearson’s *r* = 0.26, *p* = 0.22). No other characteristics/psychological measures significantly correlated with the explicit gaming desire or the IAT effect. Details are described in Table 2.

**Table 2:** Correlation coefficients between explicit gaming desire/IAT effect and characteristics/psychological measures.

	Explicit gaming desire	IAT effect
Age	*rho* = −0.17, *p* = 0.43	*rho* = 0.18, *p* = 0.38
Predicted full-scale IQ	*rho* = −0.05, *p* = 0.83	*rho* = −0.18, *p* = 0.38
Internet addiction test	*rho* = −0.11, *p* = 0.61	*rho* = −0.20, *p* = 0.33
Weekly gaming time	*rho* = −0.24, *p* = 0.24	*rho* = 0.02, *p* = 0.93
Gaming history	*r* = −0.32, *p* = 0.11	*r* = −0.18, *p* = 0.40
NEO-FFI		
Neuroticism	*r* = 0.48, *p* = 0.02*	*r* = 0.02, *p* = 0.93
Extraversion	*r* = −0.10, *p* = 0.62	*r* = −0.21, *p* = 0.30
Openness	*r* = 0.35, *p* = 0.09	*r* = −0.38, *p* = 0.06
Agreeableness	*r* = 0.07, *p* = 0.75	*r* = 0.12, *p* = 0.58
Conscientiousness	*r* = −0.11, *p* = 0.58	*r* = −0.05, *p* = 0.81
Self-efficacy	*r* = −0.06, *p* = 0.76	*r* = −0.41, *p* = 0.04*
BIS-11	*r* = 0.36, *p* = 0.08	*r* = 0.26, *p* = 0.22
CF scale	*r* = −0.14, *p* = 0.51	*r* = −0.43, *p* = 0.03*

*p < 0.05. *r* = Pearson’s correlation coefficient, rho = Spearman’s correlation coefficient. BIS-11, Barratt impulsiveness scale, 11th version; CF, cognitive flexibility; NEO-FFI, NEO-Five Factor Inventory.

## 4 Discussion

To the best of our knowledge, this is the first experimental study to investigate both the explicit and implicit effects of gaming-related content on social media among young adults. The results extend the findings of previous studies on gaming cues and may provide additional information to practically address and prevent problematic game use.

As expected, gaming-related content on social media significantly influenced the participants’ behavior explicitly and implicitly. The participants showed higher explicit gaming desire by the gaming-related cues on social media than neutral ones. They exhibited robust, positive, and implicit attitudes toward gaming-related cues on social media. Previous studies have shown that substance-related cues in digital media influence addictive behaviors of the corresponding substance. For example, exposure to alcohol marketing in social and digital media has been repeatedly reported to be associated with alcohol purchase intentions, drinking patterns, or higher levels of alcohol consumption (Lobstein et al., 2017; Noel et al., 2020). According to Chen Y. et al. (2018), their participants, regardless of smoking status, showed high neural responses to e-cigarette advertising images from online resources and exhibited behaviorally that e-cigarette advertisements made them want to smoke more than neutral advertisements. In line with these previous studies on substance-related dependence, the current results suggest that real-world online gaming-related content can elicit gaming urges; thus, this issue deserves further examination to increase our understanding of the pathogenesis of gaming addiction.

The mean response latency for the incongruent condition (game-negative and work-positive) of the IAT was significantly longer than that of the congruent condition (game-positive and work-negative). This finding indicated that the participants possessed positive implicit attitudes toward gaming-related cues on social media. The IAT reveals unconscious or implicit attitudes, automatic preferences, and hidden biases in individuals by measuring the strength of associations between concepts and evaluations (Greenwald et al., 1998, 2003). The IAT has been utilized in various areas of psychological research, such as social cognition (Birmingham et al., 2015; Fujino et al., 2017). In recent years, it has also been employed in addiction research. For example, heavy drinkers will exhibit stronger positive implicit associations toward alcohol use on the IAT compared to light drinkers (Ames et al., 2014). A recent study adapted the IAT for smartphone and internet addictions, finding it a robust and valid tool to use with children and adolescents (Roh et al., 2018). This indicates that problematic use of the internet or smartphones shares common implicit characteristics with substance-use disorders.

The explicit desire for gaming positively correlated with the levels of neuroticism. Neuroticism is a personality trait characterized by the frequent experience of negative emotions and maladaptive coping strategies, including inadequate reactions to stressful situations and interpersonal issues (Suls and Martin, 2005; Fujino et al., 2016; Marciano et al., 2020). Previous studies have identified neuroticism as an essential factor that influences problematic online gaming (Chu et al., 2022). Highly neurotic people use online social networking platforms more frequently, likely to reduce social isolation (Hamburger and Ben-Artzi, 2000; Marciano et al., 2020). Neurotic individuals may find social interactions more rewarding online than in real life when they face difficulties in real social interactions (Amichai-Hamburger et al., 2002; Tei et al., 2020). Therefore, neurotic people may be more inclined to spend time playing online games (Chu et al., 2022). Together with these previous findings, the present results suggest that individuals with high neuroticism levels can be highly affected by gaming-related content on social media, and populations susceptible to gaming addiction should receive attention.

In this study, the IAT effect scores negatively correlated with the self-efficacy levels. Self-efficacy is defined as the belief or confidence in one’s ability to cope with demands in various contexts (Bandura, 2006; Ono et al., 2018; Du and Zhang, 2022). Studies revealed that self-efficacy is relevant to internet gaming addiction like other addictions such as smoking, alcoholism, and gambling. For example, Festl et al. (2013) demonstrated that high levels of gaming addiction are linked to weaker self-efficacy in broad generations. Internet self-efficacy, which is involved in regulating one’s internet usage, is one of the significant predictors of problematic internet use (Ceyhan and Ceyhan, 2008; Shi et al., 2011). Regarding cue reactivity, previous studies have shown that a strategy focused on self-efficacy effectively reduces cue-induced craving for addictive behaviors such as smoking (Loeber et al., 2006; Ono et al., 2018). Our findings align with those of previous studies and emphasize that self-efficacy is a critical protective factor for problematic game use.

Furthermore, the participants with lower CF scores showed higher IAT effects. It has been reported that patients with IGD exhibit various types of executive dysfunction (Lee et al., 2020). Specifically, recent studies have shown that both individuals with IGD and those at risk of IGD exhibit reduced CF (Weinstein et al., 2017; Yang X. et al., 2023). Inflexible cognitive processing may produce maladaptive emotions and cognitive biases that exacerbate IGD-related behaviors (Zhou et al., 2012; Chen L. et al., 2018; Tei and Fujino, 2023). For instance, low CF can lead to poor attentional shifting and impulsive control, thereby promoting IGD-related habit formation and dereliction of social or other responsibilities (Tei et al., 2017; Weinstein et al., 2017; Király et al., 2023). However, how these CF features are associated with cue reactivity in gaming addiction is still largely unknown. Our findings will provide an impetus for future neuroimaging studies using ecologically valid cues to better understand the neuropsychological mechanisms underlying this relationship.

Previous studies have suggested that there is a connection between cue reactivity and the severity of gaming addiction (Kim et al., 2018; Ma et al., 2019). However, in this study, we did not find any significant relationships between explicit gaming desire/IAT effect and either the scores of internet addiction test or weekly gaming hours. This may be because the study participants were casual gamers from non-clinical populations, and their game use was relatively low. Therefore, this study’s major limitation is that the results cannot be generalized to individuals with IGD, and future studies should include patients with this addiction.

We found no significant correlations between explicit gaming desire/IAT effect and impulsivity. However, impulsivity is reported to play a significant role in cue reactivity, especially in the context of addiction (Cunningham and Anastasio, 2014). Impulsive individuals often exhibit heightened responsivity to environmental cues, including those related to addictive behaviors. For example, high impulsivity has been noted in cocaine-dependent individuals who express high cocaine cue reactivity (Liu et al., 2011), and a similar relationship has been observed in those of behavioral addictions including IGD (Punia and Balodis, 2019; Şalvarlı and Griffiths, 2019). Impulsivity is linked to a preference for immediate rewards over delayed gratification (Liu et al., 2011; van den Akker et al., 2014). Thus, when exposed to cues associated with addictive behaviors, impulsive individuals may be more susceptible to the immediate pleasure or relief provided by engaging in the behavior. However, relationship between cue reactivity and impulsivity is extraordinarily complex (Cunningham and Anastasio, 2014; Punia and Balodis, 2019). Thus, continued research on this topic is essential to enhance our understanding of managing cue-induced cravings and reducing the likelihood of impulsive engagement in addictive behaviors.

The current findings have practical implications for young adults increasingly exposed to gaming-related content on social media. While such content can positively foster community building, provide information updates, enhance skills, and offer entertainment, excessive exposure can impair mental and physical health (Balakrishnan and Griffiths, 2017). In response, some game developers and platforms have implemented features or mechanisms to mitigate excessive gaming and promote healthier gaming habits (Yang Q. et al., 2023). Parents, educators, and mental health professionals also promote healthy gaming habits to address potential negative impacts. As the effects of gaming-related content on social media can vary among individuals, our findings contribute to the development of a more effective and balanced approach to preventing problematic gaming use.

### 4.1 Limitations

There are several limitations to this study. First, as mentioned earlier, our sample consisted solely of young casual gamers; specifically, it did not include clinical patients with IGD or individuals who had never played online games. Therefore, caution should be exercised when interpreting our results. However, considering that IGD often emerges during adolescence or young adulthood, when individuals have increased access to online games (Aydin and Kuş, 2023; Yang Q. et al., 2023), the insights obtained from our sample of young casual gamers should offer valuable indications for improved prediction and mitigation of triggers leading to the onset of IGD.

Second, although the games featured in the stimuli were all popular online games in Japan, the varying levels of interest in these games among participants may have influenced the results. The familiarity rating scores were not significantly correlated with the levels of explicit gaming desire or the IAT effect. Nevertheless, while familiarity is a common factor shaping gaming interests (Tan et al., 2016), it can vary among individuals. Some people may develop an interest in exploring new and unfamiliar games, while others may find comfort and satisfaction in revisiting familiar titles.

Third, despite our efforts to select neutral stimuli that closely matched each gaming-related stimulus in terms of complexity, content, design, luminance, color, action, and presence of faces, the use of naturalistic stimuli for the research purpose of incorporating real-world content from social media posed challenges in achieving complete matching across multiple parameters, such as foreground-background ratio and zooming speed. This issue should be further investigated in the future studies.

Fourth, we utilized the internet addiction test, a measure designed for assessing general internet use. Internet-game-related attitudes have been reported to be more strongly related to internet addiction than other categories related to problematic internet use, such as social interaction preferences (Lee et al., 2015; Roh et al., 2018). However, the internet addiction test results may not have necessarily reflected internet-game-related attitudes of the participants.

Fifth, because of the exploratory nature of this study, no correction was applied for multiple comparisons. Thus, our preliminary findings should be interpreted cautiously, and must be carefully explored and replicated in large-scale future research.

Sixth, for the psychological measures, we utilized self-reported questionnaires. It is common to worry about false bias when self-report measures such as the NEO-FFI are used. However, we took steps to minimize the risk of false bias. We explained to participants the purpose of the study and the importance of truthful responses for obtaining valid results. We also assured participants that their responses would remain anonymous and confidential. Further details are described in the Supplementary methods.

Seventh, the sample size was small and consisted of only males. Although the sample size was comparable to previous studies using IAT (Fujino et al., 2017; Tei et al., 2018), it was relatively small for studying individual differences.

Finally, this study was conducted using a cross-sectional design, which precludes any causal conclusions. Thus, future longitudinal studies should provide additional clues to enhance our understanding of problematic game use.

## 5 Conclusion

This study found that social media content related to gaming can influence on the behavior of young adults explicitly and implicitly. Additionally, the effects of such content can differ based on the levels of neuroticism, self-efficacy, and CF among individuals. Further research using real-world gaming-related cues in digital media can help us better understand the promotion of gaming habits and support individuals vulnerable to gaming addiction.

## Data availability statement

The original contributions presented in the study are included in the article/Supplementary material, further inquiries can be directed to the corresponding author.

## Ethics statement

The studies involving humans were approved by Institutional review board of Tokyo Medical and Dental University Hospital. The studies were conducted in accordance with the local legislation and institutional requirements. The participants provided their written informed consent to participate in this study.

## Author contributions

DJ: Conceptualization, Data curation, Formal analysis, Investigation, Project administration, Writing – original draft, Writing – review & editing. NK: Conceptualization, Data curation, Formal analysis, Investigation, Writing – original draft, Writing – review & editing. YF: Conceptualization, Data curation, Investigation, Methodology, Writing – review & editing. CQ: Data curation, Investigation, Validation, Writing – review & editing. SO: Data curation, Investigation, Methodology, Writing – review & editing. ST: Methodology, Supervision, Validation, Writing – review & editing. DM: Data curation, Investigation, Supervision, Writing – review & editing. TT: Conceptualization, Investigation, Supervision, Writing – review & editing. HT: Conceptualization, Investigation, Methodology, Project administration, Supervision, Writing – review & editing. TU: Conceptualization, Funding acquisition, Investigation, Methodology, Project administration, Supervision, Writing – review & editing. MY: Conceptualization, Investigation, Methodology, Project administration, Supervision, Writing – review & editing. JF: Conceptualization, Data curation, Formal analysis, Funding acquisition, Investigation, Methodology, Project administration, Writing – original draft, Writing – review & editing.
