# Supplementary material for: Explicit and implicit effects of gaming content on social media on the behavior of young adults
Source: Front Psychol. 2024 Jan 24;15:1332462. doi: 10.3389/fpsyg.2024.1332462 (PMC10847366; doi:10.3389/fpsyg.2024.1332462)
Supplement: Supplementary file 1 [file Data_Sheet_1.docx]

Supplementary Material

**Supplementary Methods**

***Participants***

The study enrolled 26 healthy volunteers aged between 19 and 25 who played online games casually. Only male participants were selected because of a sex difference in the mechanism for gaming addiction, and men have higher gaming addiction potential than women (Dong et al., 2018, 2019; Wang et al., 2022). One participant faced a technical error during data collection, so data obtained from 25 participants were analyzed. All participants played online games regularly for at least an hour weekly and did not meet the IGD criteria proposed by the Diagnostic and Statistical Manual of Mental Disorders 5th Edition (DSM-5). They did not have any psychiatric disorders as evaluated by an experienced psychiatrist who used a structured clinical interview for DSM-5. No participants had any history of head trauma, serious medical or surgical illness, or substance abuse. Additionally, none of the participants were current smokers. To estimate the participants’ levels of gameplay, weekly gaming time and gaming history were assessed based on previous studies (Dong et al., 2018, 2019). The participants were recruited from the general population in the Tokyo area through advertisements and acquaintances. This study is a part of a larger project concerning gaming-related content on social media, and participants completed a range of measures, not all of which are reported here.

This study was approved by the institutional review board of Tokyo Medical and Dental University Hospital and was conducted in accordance with the Code of Ethics of the World Medical Association. After explaining the entire study, written informed consent was obtained from all participants.

***Study design, setting, and procedures***

This cross-sectional study occurred at Tokyo Medical and Dental University Hospital in Japan. After clinical interviews, the participants performed following behavioral tasks and psychological measures.

***Explicit desire task (EDT)***

To estimate the participants’ explicit gaming desire, we designed the EDT based on the methodologies in the previous studies of substance and behavioral addiction (Courtney et al., 2014; Dong et al., 2019; Liu et al., 2017; Vollstädt-Klein et al., 2011). Sixteen gaming-related videos such as persons enjoying a game, introducing a game, and teaching how to capture a game from social media platforms were selected. A game in each video was different from each other. The 16 games featured in the videos were all popular online games (shooting games, role-playing games, puzzle games, or sports games) in Japan. Sixteen neutral videos (nongaming videos, such as furniture-, hygiene-, travel-, and work-related videos) were also selected from social media platforms. Based on the previous studies (Chen et al., 2018; Tapert et al., 2003; Vollstädt-Klein et al., 2011), they were chosen to match as much as possible each gaming-related video on complexity, content, design, luminance, color, action, and presence of faces. Initially, we chose 220 videos from social media platforms, out of which three researchers assessed them using rating sheets that incorporated criteria related to complexity, content, design, luminance, color, action, and presence of faces. Finally, after discussing with our research team, we selected 16 neutral videos that best matched the gaming-related videos.

The videos were displayed pseudo-randomly, and after each video was presented, the participants were asked to rate the gaming desire from 1 (no desire) to 9 (extreme desire) (Fig. 2 in the main text). The experiment was conducted using E-Prime (Psychology Software Tools, Inc., Pittsburgh, PA, USA). The levels of participants’ explicit gaming desire were computed as follows: mean scores of the gaming desire in gaming-related videos–those in neutral ones. Higher scores denote higher levels of explicit gaming desire.

***Implicit association test (IAT)***

In this study, the IAT (Greenwald et al., 1998, 2003) was modified with gaming-related pictures on social media. Previous studies have revealed that the IAT reliably predicts addictive behaviors (Ames et al., 2014; Roh et al., 2018; Snagowski et al., 2015). The IAT has a series of response time tasks that require participants to classify stimuli that appear at the lower portion of the computer screen into the corresponding categories and paired attributes appearing in the upper left or right of the screen. Response times were expected to be shorter when the paired target category and attribute labels matched a person’s automatic associations. Conversely, response times are expected to be longer when the paired target category and attribute labels contradict automatic associations (Greenwald et al., 1998, 2003).

To assess individual differences in the levels of implicit positive attitudes toward gaming-related contents on social media, the IAT evaluated the extent to which participants associated game vs. work (target categories) with positive vs. negative (attribute categories) (Fig. 3 in the main text). Four items were selected as the to-be-sorted stimuli for each concept and the present IAT comprised seven blocks according to the standard procedures (Fujino et al., 2017; Greenwald et al., 1998, 2003; Tei et al., 2018). Similar to the EDT, four gaming-related pictures were captured from the social media platforms for the target concept of game. The 4 games featured in the pictures were all popular online games (shooting games, role-playing games, puzzle games, or sports games) in Japan. For the target concept of work, four work-related pictures were also selected from social media platforms. They were chosen to match as much as possible each gaming-related picture on complexity, content, design, luminance, color, action, and presence of faces. Stimuli from the categories positive/negative were happy, fun, attractive, and excited, and painful, sad, difficult, and boring, respectively. The present IAT comprised seven blocks according to the standard procedures (Greenwald et al., 1998): block 1, 20 practice trials with target categories only; block 2, 20 practice trials with attribute categories only; block 3, 20 practice trials for a congruent block with both target and attribute categories; block 4, 40 test trials for a congruent block with both target and attribute categories; block 5, 20 practice trials with target categories only in reversed positions; block 6, 20 practice trials for an incongruent block with both reversed target categories and attribute categories; and block 7, 40 test trials for an incongruent block with both reversed target categories and the attribute categories. Participants were told they would be making a series of judgments. On each trial, a stimulus was displayed at the center of a computer screen. Category labels were displayed on the left and right sides of the window. Participants used letters A and L on the left and right sides of the keyboard for their responses, respectively. They were told to be as accurate and as quickly as possible. An incorrect response resulted in the feedback of a red ‘‘×.” An inter-trial interval of 300 ms was implemented. The IAT was conducted using the E-Prime software (Psychology Software Tools, Inc.).

The IAT effect scores were computed based on the improved scoring algorithm (Greenwald et al., 2003). No participant showed a latency of <300 ms in >10% of trials. Trials with latencies >10,000 ms were deleted. Each error latency was replaced by the mean of correct latencies + 600 within the same block. The mean latency in the trials of block 3 was subtracted from that of block 6. Similarly, the mean latency in the trials of block 4 was subtracted from that of block 7. Subsequently, each difference in the score was divided by its associated trial standard deviation (SD) of response latencies. The IAT effect scores were calculated as the average of these two scores. High scores represented quicker associations of game–positive and work–negative relative (congruent condition) to game–negative and work–positive (incongruent condition); thus, higher scores indicate higher levels of implicit positive attitudes toward gaming-related contents on social media.

***IQ***

Predicted IQ was estimated using the short form of the Japanese version of the National Adult Reading Test (Matsuoka et al., 2006).

***Internet addiction test***

Based on previous studies (Dong et al., 2018, 2019), all participants completed the internet addiction test (Ishii et al., 2023; Young 1998) to measure the degree of dependence on the internet. The test measures the degree of involvement in online activities using a five-point Likert scale and classifies addictive behaviors into three categories: a total score of <40 as “no addiction,” 40–69 as “mild signs of addiction,” and ≥70 as “severe addictive behaviors.” The validity and reliability of the Japanese version of the internet addiction test were demonstrated by previous studies (Lai et al., 2015).

***Weekly gaming time***

To estimate the time participants played games, weekly gaming time was assessed based on previous studies (Dong et al., 2018, 2019)

***Gaming history***

Gaming history was defined as the difference between the current age of participants and the age at which they first started playing online games.

***Personality traits***

To assess participants’ personality traits, we used the Japanese version of the NEO-Five Factor Inventory (NEO-FFI) questionnaire (Costa and MacCrae, 1992; Shimonaka et al., 1998). This questionnaire is widely used for evaluating broad personality traits based on the five-factor model. The NEO has a high validity and test-retest reliability (Shimonaka et al., 1998), and is frequently used in studies on gaming addiction (e.g., Murray et al., 2022; Reyes et al., 2019).

The NEO-FFI is a self-report questionnaire consisting of 60 items, with each item corresponding to one of five personality trait dimensions: neuroticism, extraversion, openness, agreeableness, and conscientiousness. Participants are asked to rate their agreement with each item on a scale of 0 to 4, with 0 indicating “strongly disagree” and 4 indicating “strongly agree.”

To ensure accurate results, we provided clear instructions to participants before they completed the questionnaire, emphasizing the importance of honest and accurate responses. We also explained the purpose of the study and the significance of truthful responses. Participants’ responses were kept anonymous and confidential to encourage them to provide honest, accurate information without fear of judgment or consequences.

Furthermore, we monitored participants’ response times while completing the questionnaire to prevent excessively fast or slow response times that may indicate insufficient attention or a lack of effort in responding. The raw scores were used in statistical analyses based on previous studies (Du et al., 2002; Steinlechner et al., 2018). Higher scores indicate higher levels of a particular trait.

***Self-efficacy***

Based on the previous studies (Bandura 2006; Ono et al., 2018; Shadel et al. 2001), self-efficacy was assessed as follows: “If you encounter a situation where certain people might be tempted to play a game, how can you be sure that you would abstain from gaming? Please answer in a range from 0 (not at all) to 100 (absolutely sure).” A single-item measure of self-efficacy has been reported to be reliable and valid (Bandura 2006; Ono et al., 2018; Shadel et al. 2001). Higher scores indicate higher levels of self-efficacy.

***Impulsivity***

Impulsivity was assessed by the Japanese version of the Barratt Impulsiveness Scale, 11th version (BIS-11) (Patton et al., 1995; Someya et al., 2001). The BIS-11 contains a total of 30 items, and each item is answered on a 4-point Likert scale. Higher scores indicate higher impulsivity.

***CF***

To estimate CF abilities, the Japanese version of the CF scale (12 items) that examines an individual’s awareness of alternative ways of behaving and the willingness and perceived ability to be flexible (Martin and Rubin, 1995; Oshiro et al., 2016) was employed. This scale has been applied in many previous studies, and studies have reported its usefulness for studying impairment of this ability in various psychiatric disorders (Sami et al., 2023; Fujino et al., 2017, 2019). Each item is rated on a 6-point Likert scale. Higher scores indicate greater CF abilities.

**Supplementary References**

Ames, S. L., Grenard, J. L., He, Q., Stacy, A. W., Wong, S. W., Xiao, L., et al. (2014). Functional imaging of an alcohol‐Implicit Association Test (IAT). Addiction Biology, 19(3), 467-481. DOI: 10.1111/adb.12071

Bandura, A. (2006). Guide for constructing self-efficacy scales. Self-efficacy Beliefs of Adolescents, 5(1), 307-337.

Chen, Y., Fowler, C. H., Papa, V. B., Lepping, R. J., Brucks, M. G., Fox, A. T., et al. (2018). Adolescents' behavioral and neural responses to e-cigarette advertising. Addiction biology, 23(2), 761-771. DOI: 10.1111/adb.12510

Costa, P. T., & McCrae, R. R. (1992). Professional Manual for Revised NEO Personality Inventory. Odessa, Fla. Psychological Assessment Resources.

Courtney, K. E., Ghahremani, D. G., London, E. D., & Ray, L. A. (2014). The association between cue-reactivity in the precuneus and level of dependence on nicotine and alcohol. Drug and alcohol dependence, 141, 21-26. DOI: 10.1016/j.drugalcdep.2014.04.026

Dong, G., Wang, L., Du, X., & Potenza, M. N. (2018). Gender-related differences in neural responses to gaming cues before and after gaming: Implications for gender-specific vulnerabilities to Internet gaming disorder. Social cognitive and affective neuroscience, 13(11), 1203-1214. DOI: 10.1093/scan/nsy084

Dong, G., Wang, Z., Wang, Y., Du, X., & Potenza, M. N. (2019). Gender-related functional connectivity and craving during gaming and immediate abstinence during a mandatory break: Implications for development and progression of internet gaming disorder. Progress in Neuro-Psychopharmacology and Biological Psychiatry, 88, 1-10. DOI: 10.1016/j.pnpbp.2018.04.009

Du, L., Bakish, D., Ravindran, A. V., & Hrdina, P. D. (2002). Does fluoxetine influence major depression by modifying five-factor personality traits?. Journal of Affective Disorders, 71(1-3), 235-241. DOI: 10.1016/s0165-0327(01)00370-6

Fujino, J., Tei, S., Itahashi, T., Aoki, Y., Ohta, H., Kubota, M., et al. (2019). Need for closure and cognitive flexibility in individuals with autism spectrum disorder: A preliminary study. Psychiatry Research, 271, 247-252. DOI: 10.1016/j.psychres.2018.11.057

Fujino, J., Tei, S., Jankowski, K. F., Kawada, R., Murai, T., & Takahashi, H. (2017). Role of spontaneous brain activity in explicit and implicit aspects of cognitive flexibility under socially conflicting situations: a resting-state fMRI study using fractional amplitude of low-frequency fluctuations. Neuroscience, 367, 60-71. DOI: 10.1016/j.neuroscience.2017.10.025

Greenwald AG, McGhee DE, Schwartz JL (1998). Measuring individual differences in implicit cognition: the implicit association test. Journal of Personality and Social Psychology 74:1464–1480. DOI: 10.1037//0022-3514.74.6.1464

Greenwald AG, Nosek BA, Banaji MR (2003). Understanding and using the implicit association test: I. An improved scoring algorithm. Journal of Personality and Social Psychology 85:197–216. DOI: 10.1037/0022-3514.85.2.197

Ishii, S., Takagi, S., Kobayashi, N., Jitoku, D., Sugihara, G., & Takahashi, H. (2023). Hyperfocus symptom and internet addiction in individuals with attention-deficit/hyperactivity disorder trait. Frontiers in Psychiatry, 14, 1127777. DOI: 10.3389/fpsyt.2023.1127777

Lai, C. M., Mak, K. K., Cheng, C., Watanabe, H., Nomachi, S., Bahar, N., et al. (2015). Measurement invariance of the internet addiction test among Hong Kong, Japanese, and Malaysian adolescents. Cyberpsychology, Behavior, and Social Networking, 18(10), 609-617. DOI: 10.1089/cyber.2015.0069

Liu, L., Yip, S. W., Zhang, J. T., Wang, L. J., Shen, Z. J., Liu, B., et al. (2017). Activation of the ventral and dorsal striatum during cue reactivity in Internet gaming disorder. Addiction biology, 22(3), 791-801. DOI: 10.1111/adb.12338

Martin, M. M., & Rubin, R. B. (1995). A new measure of cognitive flexibility. Psychological Reports, 76(2), 623-626. DOI:10.2466/pr0.1995.76.2.623

Matsuoka, K., Uno, M., Kasai, K., Koyama, K., & Kim, Y. (2006). Estimation of premorbid IQ in individuals with Alzheimer’s disease using Japanese ideographic script (Kanji) compound words: Japanese version of National Adult Reading Test. Psychiatry and Clinical Neurosciences, 60(3), 332-339. DOI: 10.1111/j.1440-1819.2006.01510.x

Murray, A., Mannion, A., Chen, J. L., & Leader, G. (2022). Gaming disorder in adults with autism spectrum disorder. Journal of Autism and Developmental Disorders, 52, 2762-2769. DOI: 10.1007/s10803-021-05138-x

Ono, M., Kochiyama, T., Fujino, J., Sozu, T., Kawada, R., Yokoyama, N., et al. (2018). Self‐efficacy modulates the neural correlates of craving in male smokers and ex-smokers: an fMRI study. Addiction biology, 23(5), 1179-1188. DOI: 10.1111/adb.12555

Oshiro, K., Nagaoka, S., & Shimizu, E. (2016). Development and validation of the Japanese version of cognitive flexibility scale. BMC Research Notes, 9(1), 1-8. DOI: 10.1186/s13104-016-2070-y

Patton, J. H., Stanford, M. S., & Barratt, E. S. (1995). Factor structure of the Barratt impulsiveness scale. Journal of Clinical Psychology, 51(6), 768-774. DOI: 10.1002/1097-4679(199511)51:6<768::aid-jclp2270510607>3.0.co;2-1

Reyes, M. E. S., Davis, R. D., Lim, R. A. N. N., Lim, K. R. S., Paulino, R. F., Carandang, A. M. D., & Azarraga, M. G. S. (2019). Five-factor model traits as predictors of pathological gaming among selected Filipino gamers. Psychological Studies, 64, 213-220. DOI:10.1007/s12646-019-00498-y

Roh, D., Bhang, S. Y., Choi, J. S., Kweon, Y. S., Lee, S. K., & Potenza, M. N. (2018). The validation of Implicit Association Test measures for smartphone and Internet addiction in at-risk children and adolescents. Journal of Behavioral Addictions, 7(1), 79-87. DOI: 10.1556/2006.7.2018.02

Sami, H., Tei, S., Takahashi, H., & Fujino, J. (2023). Association of cognitive flexibility with neural activation during the theory of mind processing. Behavioural Brain Research, 443, 114332. DOI: 10.1016/j.bbr.2023.114332

Shadel WG, Niaura R, Goldstein MG, Abrams DB (2001). Cognitive avoidance as a method of coping with a provocative smoking cue: the moderating effect of nicotine dependence. Journal of Behavioral Medicine 24:169–182. DOI: 10.1023/a:1010762631464

Shimonaka, Y., Nakazato, K., Gondo, Y. & Takayama, M. (1998). Construction and factorial validity of the Japanese NEO-PI-R. The Japanese Journal of Personality 6, 138–147. DOI: 10.2132/jjpjspp.6.2_138

Snagowski, J., Wegmann, E., Pekal, J., Laier, C., & Brand, M. (2015). Implicit associations in cybersex addiction: Adaption of an Implicit Association Test with pornographic pictures. Addictive Behaviors, 49, 7-12. DOI: 10.1016/j.addbeh.2015.05.009

Someya, T., Sakado, K., Seki, T., Kojima, M., Reist, C., Tang, S. W., et al. (2001). The Japanese version of the Barratt Impulsiveness Scale, 11th version (BIS-11): Its reliability and validity. Psychiatry and clinical neurosciences, 55(2), 111-114. DOI: 10.1046/j.1440-1819.2001.00796.x

Steinlechner, S., Jabusch, H. C., Altenmüller, E., Borngräber, F., Hagenah, J., Klein, C., et al. (2018). Personality profiles are different in musician's dystonia and other isolated focal dystonias. Psychiatry Research, 266, 26-29. DOI: 10.1016/j.psychres.2018.05.017

Tapert, S. F., Cheung, E. H., Brown, G. G., Frank, L. R., Paulus, M. P., Schweinsburg, A. D., et al. (2003). Neural response to alcohol stimuli in adolescents with alcohol use disorder. Archives of general psychiatry, 60(7), 727-735. DOI: 10.1001/archpsyc.60.7.727

Tei, S., Fujino, J., Hashimoto, R. I., Itahashi, T., Ohta, H., Kanai, C., et al. (2018). Inflexible daily behaviour is associated with the ability to control an automatic reaction in autism spectrum disorder. Scientific reports, 8(1), 8082. DOI: 10.1038/s41598-018-26465-7

Vollstädt-Klein, S., Kobiella, A., Bühler, M., Graf, C., Fehr, C., Mann, K., & Smolka, M. N. (2011). Severity of dependence modulates smokers' neuronal cue reactivity and cigarette craving elicited by tobacco advertisement. Addiction biology, 16(1), 166-175. DOI: 10.1111/j.1369-1600.2010.00207.x

Wang, L., Zheng, H., Wang, M., Chen, S., Du, X., & Dong, G. H. (2022). Sex differences in neural substrates of risk taking: Implications for sex-specific vulnerabilities to internet gaming disorder. Journal of Behavioral Addictions, 11(3), 778-795. DOI: 10.1556/2006.2022.00057

Young KS. (1998). Caught in the Net: How to Recognize the Signs of Internet Addiction- and a Winning Strategy for Recovery. New York: Wiley.
